# Automatic couch position calculation using eclipse scripting for external beam radiotherapy

**DOI:** 10.1002/acm2.13159

**Published:** 2021-01-13

**Authors:** Hesheng Wang, Anthony Rea, Benedikt Rudek, Ting Chen, Allison McCarthy, David Barbee

**Affiliations:** ^1^ Department of Radiation Oncology NYU Langone Health NYU Grossman School of Medicine New York University New York NY USA

**Keywords:** ESAPI, patient safety, treatment couch position, wrong‐site error

## Abstract

**Purpose:**

The treatment couch position of a patient in external beam radiation therapy (EBRT) is usually acquired during initial treatment setup. This procedure has shown potential failure modes leading to near misses and adverse events in radiation treatment. This study aims to develop a method to automatically determine the couch position before setting up a patient for initial treatment.

**Methods:**

The Qfix couch‐tops (kVue and DoseMax) have embedded reference marks (BBs) indicating its index levels and couch centerline. With the ESAPI, a C# script was programmed to automatically find the couch‐top and embedded BBs in the planning CT and derive the treatment couch position according to treatment isocenter of a plan. Couch positions of EBRT plans with the kVue couch‐top and SBRT plans using the DoseMax were calculated using the script. The calculation was evaluated by comparing calculated positions with couch coordinates captured during the initial treatment setup after image guidance. The calculations were further compared with daily treatment couch positions post image‐guided adjustment for each treatment fraction.

**Results:**

For plans using the kVue couch‐top for various treatment sites, the median (5–95 percentiles) differences between calculated and captured couch positions were 0.1 (−0.2 – 0.9), 0.5 (−1.1–2.0), 0.10 (−1.3–1.3) cm in the vertical, longitudinal, and lateral direction respectively. For the DoseMax couch‐top, the median differences were 0.1 (−0.2–0.7), 0.2 (−0.3–1.1), and 0.2 (−0.7–0.9) cm in respective direction. The calculated positions were within 1 and 2 cm from the mean fraction positions for 95% patients on DoseMax and kVue couch‐top respectively.

**Conclusions:**

A method that automatically and accurately calculates treatment couch position from simulation CT was implemented in Varian Eclipse for Qfix couch‐tops. This technique increases the efficiency of patient setup and enhances patient safety by reducing the risks of positioning errors.

## 1 INTRODUCTION

Setting up a patient to the same position as planned is crucial for safe and accurate delivery of radiotherapy. The planned treatment couch position is commonly specified as shifts in vertical (anterior/posterior), longitudinal (superior/inferior), and lateral (left/right) directions relative to triangulation or biangulation fiducial markers that are tattooed during CT simulation. On the day of treatment, therapists first position the patient on the treatment couch by aligning the skin tattoos to in‐room lasers, and then move the couch according to the planned shifts in each of the three directions. Typically, MV portal filming and/or radiographic 2D setup projections are subsequently performed for patient position verification and correction. Tumor localization and patient position may be further assessed by cone‐beam CT (CBCT) and/or nonradiographic techniques.^1^ The common clinical practice is to capture the couch coordinates after image approval on the first treatment day. The record‐and‐verify (R&V) system uses the coordinates as a baseline, in conjunction with tolerance tables, to ensure proper positioning in subsequent fractions of treatment delivery, providing the first line of defense against patient setup error.

Geometric miss due to incorrect patient setup is one of the prominent causes for radiotherapy incidents. The treatment couch position is an important clinical parameter to prevent gross setup errors. However, the position captured at the initial treatment setup becomes erroneous when the couch shifts are wrongly instructed in the treatment plan or incorrectly applied during setup. For example, the planners mistakenly identify fiducial markers or lines on the simulation CT for triangulation points, and the therapists misinterpret couch shift instructions or align in‐room lasers with the skin tattoos from previous treatment. Analysis of the Radiation Oncology Incident Learning System (RO‐ILS) showed that 74 of the 396 events resulted from either wrong shift instructions or wrong shifts performed.^2^ Gross treatment‐site errors usually can be detected by the following imaging verification. However, recognizing a setup error typically initiates a root‐cause investigation, which increases the time for patient on the table and puts pressures on therapists and physicists. Additionally, the setup errors could be missed on setup images due to insufficient training of the therapists, such as wrong identification of vertebral levels in spinal irradiation.^3^

Given the fact that the captured couch position is subject to human errors, an automatic determination of the treatment couch position in advance could eliminate associated failure modes and mitigate potential pressures on the team during patient setup. In current clinical workflow of radiation treatment, the majority of patients are CT simulated and LINAC treated with the same immobilization on identical indexed treatment couches. The indexing builds a one‐to‐one correspondence from CT space to treatment coordinates, allowing the derivation of treatment couch position from simulation CT images before the initial treatment setup. Using this correspondence, Saenz et al.^4^ predicted the couch position based on the radio‐opaque landmark on immobilization devices. Instead, treatment couch coordinates were estimated from the couch either based on its embedded ball bearings (BBs) by Tsai et al.^5^ or indexing notches by Sueyoshi et al.^6^ Both methods involved manual selection of a point (a reference BB^5^ or the user origin^6^) on CT images during treatment planning, which is potentially subjected to user error and alters the planning workflow. In light of this, we developed an automated solution to determine the treatment couch position by computerized detection of the embedded BBs and index levels on the couch from simulation CT images. Moreover, we implemented the method as a scripting function that seamlessly integrates with the treatment planning system for efficient clinical utilization.

## 2 METHODS AND MATERIALS

### 2.A Qfix couch‐top treatment position calculation

**Fig. 1:**
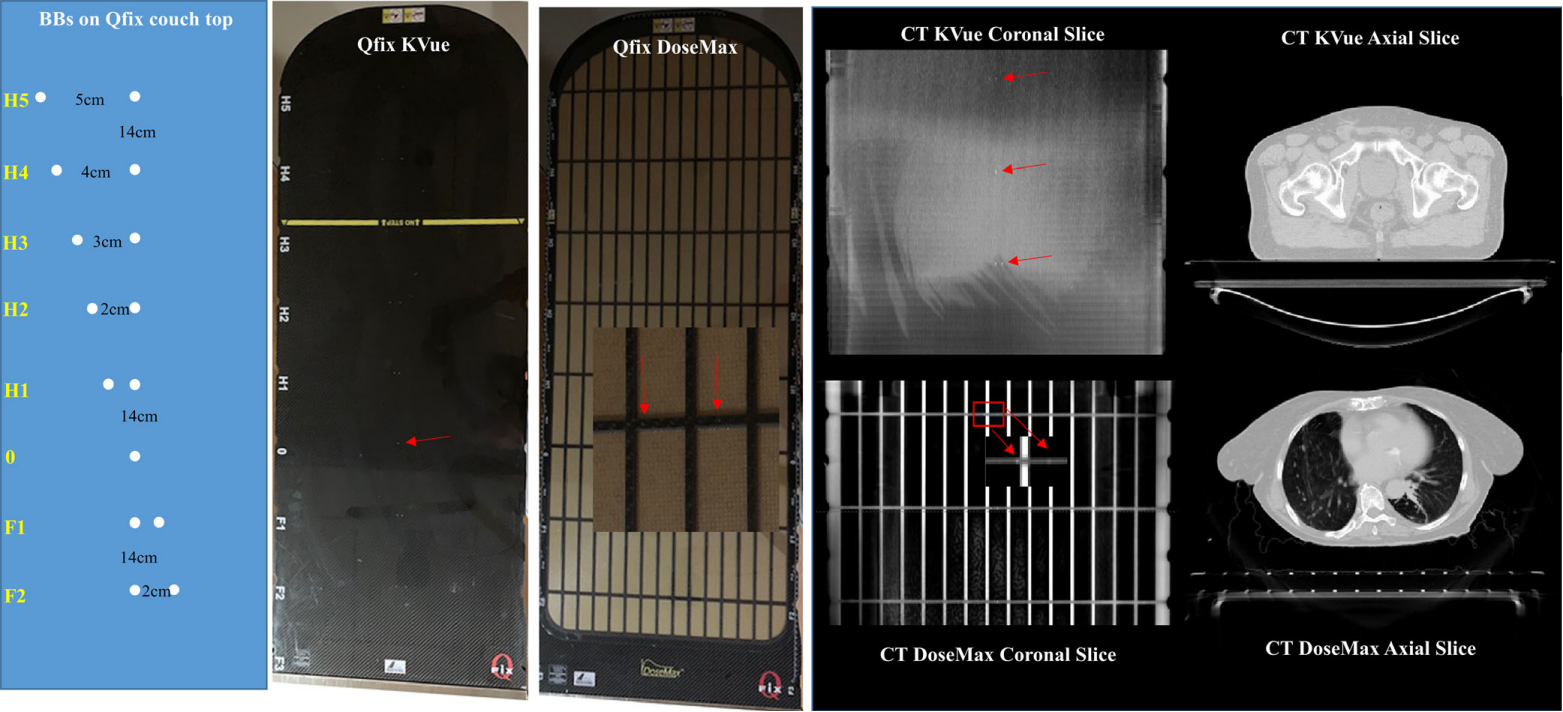
Qfix kVue and DoseMax couch‐top. From left to right: scheme of BBs on a couch‐top, Qfix kVue couch‐top, DoseMax couch‐top, computed tomography coronal and axial images for both couch‐tops.

The majority of patients receiving radiotherapy at our institution are CT simulated and LINAC treated on a Qfix couch‐top (Qfix, Avondale, PA). The Qfix kVue couch‐top is used for conventional EBRT, while the Qfix DoseMax couch‐top is utilized for SBRT. Incorporating their Virtual Indexing^TM^ technique, both Qfix couch‐tops have embedded radio‐opaque BBs that are distributed on the couch surface in lateral rows with a 14cm longitudinal interval (Fig. 1). From the head downwards, the BB rows are labeled as index levels H5 to H1, 0, F1, F2, and/or F3 at the side along with the notches for fixing immobilization devices. At each row, one BB is located at the couch midline, and a second BB is laterally seated to the right for a F‐Index and to the left for a H‐Index. The distance between two BBs in a row corresponds to the index level (e.g. 4 cm distance for the H4‐Index). The row of 0‐index only has a single BB at the midline.

Following the International Electrotechnical Commission standard (IEC61217), the treatment couch is calibrated at the lateral (*X*), vertical (*Y*), and longitudinal (*Z*) position (*TX_0_, TY_0_, TZ_0_*) of 0, 0, and 140 cm, respectively, when the couch BB at the 0‐Index (*reference BB*) is aligned with the machine isocenter. With the coordinates of planning treatment isocenter (*Xiso, Yiso, Ziso*) and reference BB (*X_0_, Y_0_, Z_0_*) in the TPS, the couch position at treatment (*TX, TY, TZ*) is calculated by.

*TX* = *X_0_* – *Xiso* + *TX_0_*,
*TY* = *Y_0_* – *Yiso* + *TY_0_*,
*TZ* = *Z_0_* – *Ziso* + *TZ_0_*

As the equations show, as long as the coordinate *(X_0_, Y_0_, Z_0_)* of the reference BB is decided from CT images, the treatment couch position can be calculated immediately when the treatment isocenter is specified during the planning. While (*TX_0_, TY_0_, TZ_0_*) for the reference 0‐Index BB is commonly set to (0, 0, 140) for Varian LINACs, the principle is also valid for calculations of treatment couch positions calibrated in other ways.

### 2.B Computerized detection of the reference BB

**Fig. 2:**
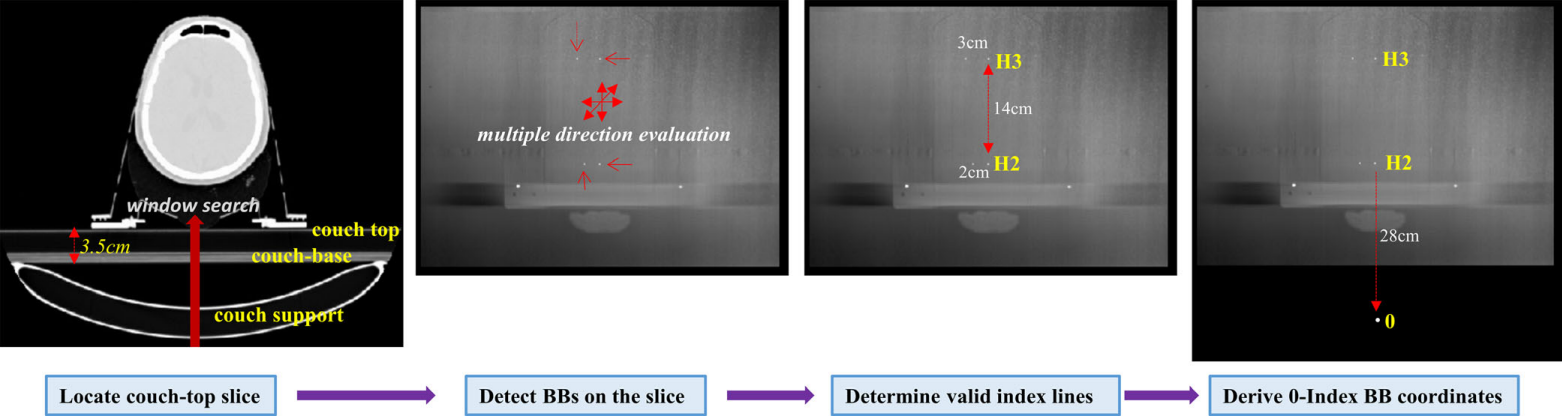
Workflow of automatic couch position calculation from simulation computed tomography.

Our method first searches for the coronal slice of the couch‐top in the axial simulation CT volume and then detects BBs on the slice using pre‐defined CT thresholds. Depending on the treatment site, the CT longitudinal scanning range may not include the reference BB at the 0‐Index level. This method thus derives the index levels for detected BB rows, and subsequently estimates the reference BB coordinates by extrapolating the CT to the 0‐Index line. Finally, the treatment couch position is calculated using the equation above. Figure 2 shows the workflow for reference BB detection.

The CT slice of a couch‐top is searched according to the mean CT number of a central window on 2D coronal slices of the 3D CT volume. The search is performed vertically from the bottom slice (Fig. 2) until reaching the couch‐base slice with a mean CT number greater than a couch‐base CT threshold (−600). The algorithm further tests CT values for the next couch‐base slice and an air slice in the middle between couch base and top. However, searching from the bottom often meets the high‐density couch support arcs before the couch base. Additional simultaneous checks of CT numbers laterally away from the central window are performed to ensure a flat couch base with relatively uniform CT numbers. The vertical coordinate *Y_0_* of the couch‐top surface is then obtained by adding the distance from the couch base to top which is constant (3.5 cm for both Qfix kvue and DoseMax couch‐tops).

BB detection is then performed on the found 2D coronal couch‐top slice. The first BB is identified by testing each pixel 5mm laterally from the slice midline. The other BB in the same row is searched on pixels every 1cm away from the first BB with a maximum distance of 5cm since the maximum index level is H5 (5cm laterally between the two BBs). Subsequently, the BB detection moves to the row in 14cm longitudinal distance until the whole coronal slice is assessed. The BB test on a pixel checks that its CT number is greater than a BB CT threshold (−350) and the size of the local‐maximum region is less than 3 pixels in any direction (multiple direction evaluation in Fig. 2). The test is also conducted on the pixel in the slices immediately below and above the couch‐top slice to account for possible tilt of the couch‐top in a CT scan. The pixel passing the BB test and having the greatest contrast in the three slices is chosen as the coordinate of a couch‐top BB.

The index level for a BB row is determined based on the direction and distance of the side BB to the medial one. The method calculates the longitudinal distances between BB rows using their CT coordinates, and compares the distance with the presumptive separation based on the determined index levels. The matching test selects a valid BB row to account for missing BBs in detection due to reduced CT contrast resulting from partial volume effect in the voxel. Finally, the coordinates *(X_0_, Y_0_, Z_0_)* of the reference BB at the 0‐Index is estimated from the valid row midline BB and its index level.

### 2.C ESAPI scripting implementation

The method was implemented using C# script with the Eclipse Scripting Application Programming Interface (ESAPI) which is integrated with the Eclipse treatment planning system (TPS) (Eclipse 15.6, Varian Medical Systems, Palo Alto, CA). ESAPI provides access to CT and planning data of the patient loaded in Eclipse and offers tools to transform between CT coordinates and positions in the TPS user space. The script is run as an add‐on in the TPS to provide the treatment couch position when a treatment isocenter is defined on the simulation CT images.

### 2.D Clinical verification

The method was verified on patient data for 55 SBRT with the Qfix DoseMax couch‐top and 122 conventional EBRT patients on the Qfix kVue couch‐top. All patients were simulated on Siemens SOMATOM CT scanners (Siemens Medical Solution, Malvern, PA) and treated on Varian TrueBeam or Edge LINACs with 6 degree‐of‐freedom (DOF) couches using the same couch‐top and indexing for immobilization devices. The treatment couch position was captured immediately after MV/KV planar imaging‐guided patient setup at the initial treatment day (the setup procedure day or the first treatment day), and then used as the baseline position for subsequent treatment. If prescribed, CBCT was also acquired for tumor localization and patient positioning prior to irradiation. However, only translational shifts of the resulting couch parameters are captured because 6DOF couch positions cannot be acquired due to the angular components (rotation, pitch, and roll). Nevertheless, the 6DOF corrections are utilized for daily treatment if CBCT guidance prescribed. The full daily treatment couch positions post daily image guidance were recorded in the R&V system on each treatment day.

Performance of the BB detection was first evaluated by comparing the calculated BB coordinates with the BB positions manually selected on CT images in the TPS. Afterwards, calculated positions and captured couch coordinates were compared for the differences in each direction. Accuracy of the automatic calculation method was assessed for various treatment sites. Moreover, the isocenter translational coordinates of the couch positions at each fraction were extracted from the recorded daily 6DOF positions. The calculated couch positions were compared with means of the daily couch parameters to evaluate the feasibility of using the computed position as the baseline couch parameter for the entire treatment course.

## 3 RESULTS

Couch‐top BBs were manually selected on simulation CTs of 41 plans using the kVue couch‐top and 22 plans with the DoseMax table‐top. Regardless of immobilization devices or treatment sites, the differences between the coordinates of automatic detected BBs and manual selections were within 0.1 cm in each direction on both couch‐tops. Means of absolute differences (mean±std) between the two coordinates were 0.04 ± 0.05, 0.06 ± 0.05, 0.02 ± 0.04 cm in the vertical, longitudinal, and lateral direction respectively.

**Fig. 3:**
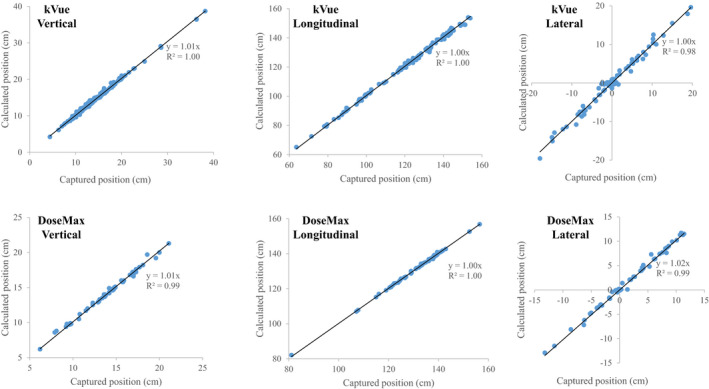
Scatter plots of captured vs calculated couch positions in three directions for the kVue and DoseMax couch‐tops.

Figure 3 plotted the linear relationships between captured and calculated couch positions for both couch‐tops in a wide range of treatment couch coordinates. In the 122 plans using the kVue couch‐top, the median (5 – 95 percentiles) differences between the two positions were 0.1 (−0.2–0.9), 0.5 (−1.1–2.0), 0.10 (−1.3–1.3) cm in the vertical, longitudinal, and lateral direction, respectively. The median differences were 0.1 (−0.2–0.7), 0.2 (−0.3–1.1), and 0.2 (−0.7–0.9) cm in respective direction for the 55 SBRT plans with the DoseMax couch‐top.

**Fig. 4:**
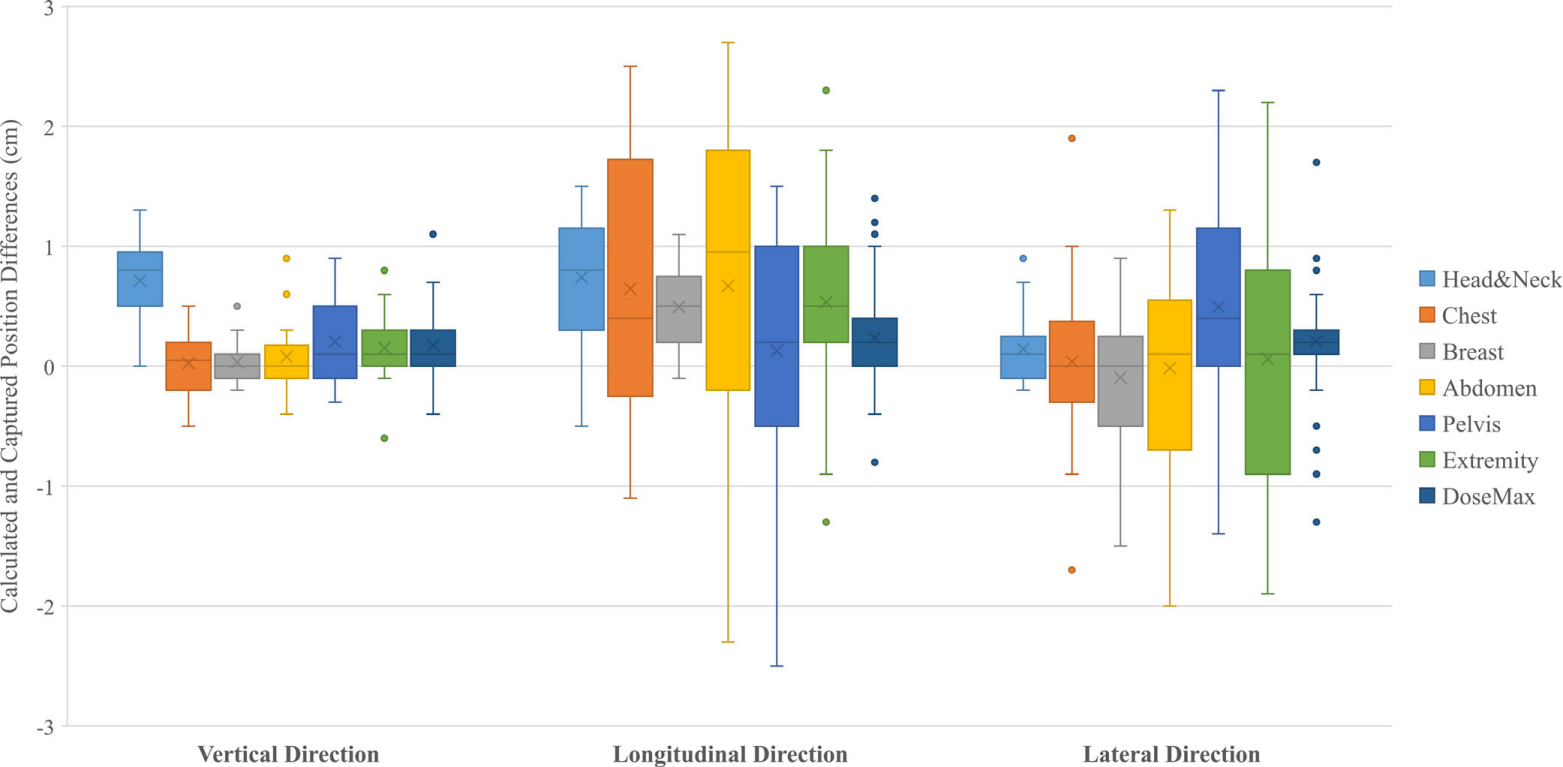
Whisker box plots of the differences between calculated and captured couch positions for different treatment sites using kVue couch‐top. The position differences of all plans on a DoseMax table was also included.

Figure 4 showed the differences between calculated and captured kVue couch positions based on treatment sites including 21 head and neck, 20 chest, 17 breast, 20 abdomen, 21 pelvis and 23 extremity. A systematic shift was observed in the longitudinal direction, likely due to the compensation for treatment couch sag under patient load. The position differences of the DoseMax couch‐top for various sites exhibited the least deviation between calculation and acquisition in all three directions. The patients on a DoseMax couch‐top undertaking SBRT were constrained in custom molds created by Vac‐loc bags, forming highly reproducible patient setup. In comparison, patients positioning on the kVue couch‐top using immobilization devices such as prone or supine breast boards for breast, a shutter board for chest, and a belly board for pelvis patients were less reproducible. Therefore, substantial deviations between calculated and captured couch positions were observed in the longitudinal direction for the sites of chest and abdomen.

**Fig. 5:**
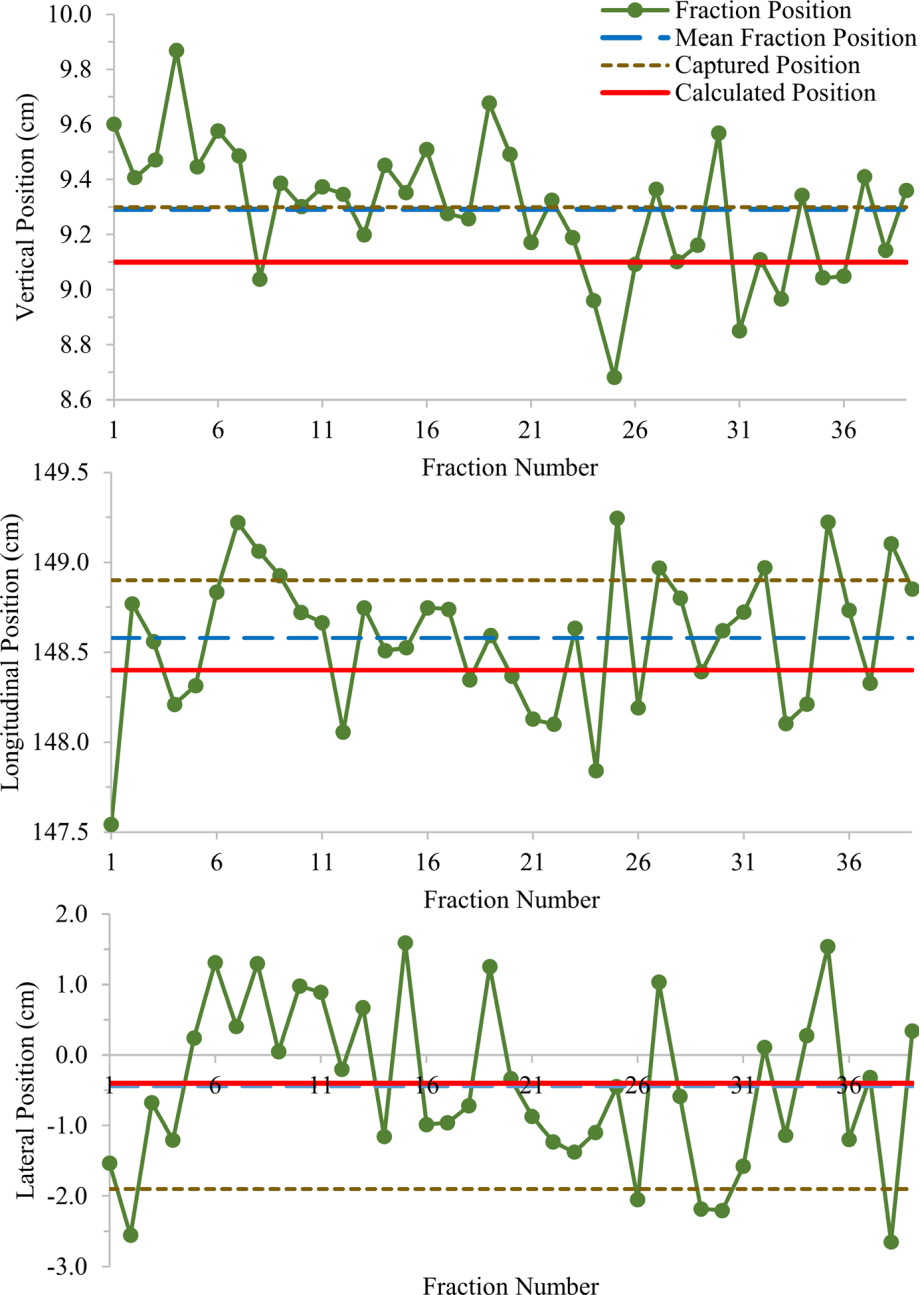
Plots of daily fraction couch position, mean fraction position, captured and calculated positions of a patient example receiving 39 fraction radiotherapy for prostate. The captured couch positions were obtained in the setup procedure day.

The fraction couch positions of a patient example (Fig. 5) demonstrated the substantial interfraction uncertainty of patient positioning during a multiple fraction treatment. The 39‐fraction prostate‐bed irradiation had fraction couch position ranges (max ‐ min) of 1.2, 1.7, and 4.2 cm in the vertical, longitudinal, and lateral direction respectively. The calculated couch position had differences of −0.2, −0.2, and 0.0 cm from the mean fraction position in the same directions, potentially being a valid choice for the baseline couch position in the treatment course.

**Fig. 6:**
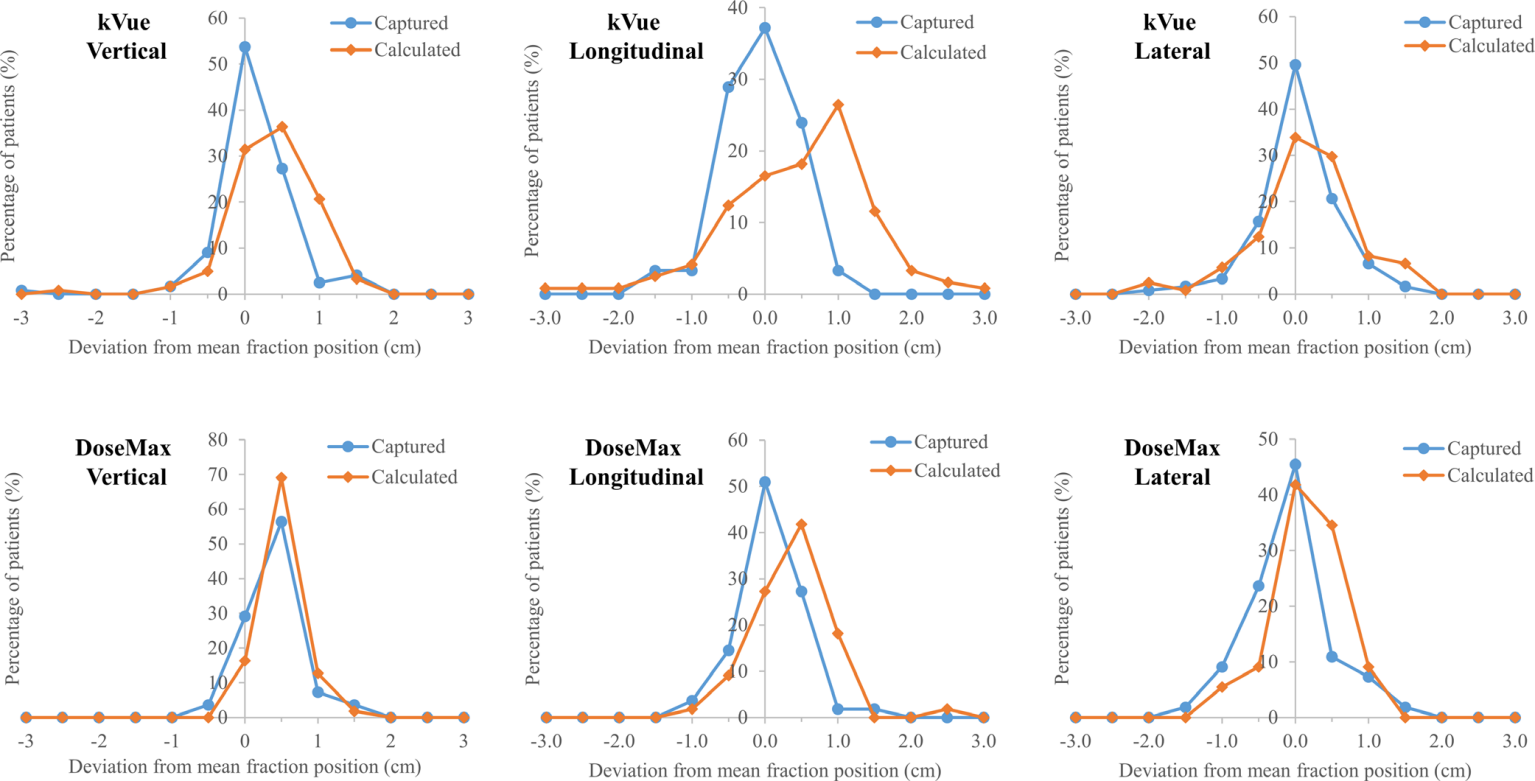
Patient histograms on distributions of the deviations between calculated, captured couch positions and the mean fraction positions.

The numbers of total treatment fractions were 1797 and 259 for patients on the kVue and DoseMax couch‐top, respectively. The deviations of the captured and calculated couch positions from the mean fraction positions were computed for each patient. The distributions of the deviations were shown in normalized patient histograms in Fig. 6. With the DoseMax couch‐top, the deviations on 96% patients were within 1cm for both the captured and calculated couch positions in either direction. For the kVue table‐top, 95% of patient captured couch positions had less than 1cm distances from the mean fraction positions. However, for the calculated couch positions, patients with <1 cm deviation in the vertical, longitudinal, and lateral direction were 95%, 78%, and 90%, respectively, which became 96% in all three directions for deviation <2 cm. The spreading of the histograms for the calculated couch positions appeared wider than those of the captured positions, suggesting that the captured couch position seems better to account for the setup variation from simulation to treatment.

## 4 DISCUSSIONS

A number of failure modes leading to wrong‐site treatment errors occur in shifting a treatment couch to the planned position during patient setup.^2^ In this work, we developed a method that automatically calculates the treatment couch position from the simulation CT image before actual patient positioning at the first treatment day. The method was implemented as an add‐on function to the TPS, allowing seamless integration with current radiotherapy workflow. The calculated position can be entered into the R&V system to directly set the couch for treatment or to verify the couch parameter after common tattoo‐based shifts. Clinical utilization of the tool could improve the efficiency and safety in EBRT treatment delivery.

The basis to estimate treatment couch position is using landmarks identifiable on simulation CT and knowledge of the couch coordinates when the landmark is aligned with machine isocenter. Saenz et al.^4^ used CT‐apparent landmarks on an immobilization device. A user manually selected the landmarks on CT images, and calculated treatment couch position based on predetermined couch coordinates for the landmarks. The dependence on immobilization devices with radiographic landmarks limited the clinical use of the method. The recent method manually selected a couch‐top BB on the CT images and referred it to another BB with known couch coordinate for couch position calculation.^5^ Sueyoshi et al.^6^ manually chose a couch indexing notch for longitudinal reference and placed the user origin at the midline of the CT couch surface during treatment planning. Both of the methods need the user to specify a selected reference in a program outside the TPS for couch coordinate estimation. Our method automatically determines the couch‐top BBs and indexing levels on CT images, and provides the treatment couch position inside the TPS without manual interventions. Thereby, this method reduces the risk of user‐related adverse events and requires the least changes to the daily treatment planning workflow.

The feasibility and accuracy of estimating treatment couch position based on the CT couch‐top has been demonstrated in previous studies ^5^, ^6^ as well as this study. Positioning the treatment couch‐top at the calculated coordinates has been used on EBRT of approximate 300 patients without wrong‐site treatment errors.^6^ Compared with the couch coordinates captured on the initial treatment day, the absolute differences of the calculated positions of our results had mean (mean±std) of 0.3 ± 0.3, 0.7 ± 0.6, 0.5 ± 0.5 cm in vertical, longitudinal, and lateral direction on both couch‐tops with various treatment sites and immobilization devices. As the couch positions were captured after MV/KV planar imaging‐based, bony structure‐focused patient positioning, these deviations were largely due to the changes in patient posture and position from simulation to treatment. When CBCT imaging was used for tumor localization and soft tissue‐based verification, the discrepancies between calculated and fraction couch positions also subjected to patient internal anatomy changes. While the interfraction internal changes could be substantial, Fig. 6 demonstrated that the calculated couch position could be valid as a safety precaution against gross positioning error.

The script calculated the couch coordinates when a treating patient was exactly in the planning position characterized in the simulation CT. However, the interfraction variations of patient position and internal anatomy are inherent to radiation treatment. In EBRT, An R&V system monitors actual treatment couch position vs the baseline coordinate with a tolerance table, preventing gross setup error during whole treatment course. A common practice is to use the captured position at initial treatment as such baseline. The script‐calculated couch position enables the safety measure for the 1st fraction, but also could be applicable for the remaining fractions. Appropriate population‐based site‐specific tolerance tables need be established for this application.^7^ However, while the calculated position is an option for the baseline couch parameter, the captured couch coordinates seem to better represent the average couch position during the entire treatment course. Nevertheless, subsequent image‐guided tumor localization and patient positioning should determine the final couch position for daily treatment.

The threshold‐based BB detection on CT couch‐top slices may fail when its CT contrast is substantially reduced due to partial volume effects from a small couch tilt and/or proximity to a high‐density immobilization device. The method checked the validity of a detected BB by testing that the longitudinal distance between two BB rows calculated from their CT coordinates match with that derived from their index levels. A warning is reported if only a single index line or no valid BB row is found on the CT images. In these cases, or as an independent second check, a user can manually select a BB line on CT images, determine the index level, and manually calculate the couch position in the way the script does. An in‐house software could implement the computation logic based on the inputs of a central BB coordinate, corresponding index level, and treatment isocenter position.^5^

Similar to previously reported CT‐based approaches,^4^, ^5^, ^6^ our method relies on that the patient is set up on a treatment couch with exactly identical indexing of the same immobilization device as in the simulation. Incorrect immobilization or indexing lead to deviation of the actual treatment table position from the calculated values, which could alert therapists for correction. However, if the setup inconsistencies are necessary for gantry clearance or other reasons, the calculated couch positions could be updated according to the actual indexing and immobilization setting. Additionally, this method is not applicable for nonindexing patient setups, e.g. clinical electron setup, or a couch‐insert without embedded landmark BBs.

Patient setup using predetermined couch position and subsequent image guidance enables markless isocenter localization without the need of skin tattoos.^6^ The permanent tattoos for conventional radiotherapy could have significant psychosocial impacts on patients, especially breast cancer patients and pediatric patients.^8^, ^9^ The implemented method may be useful for tattoo‐less radiation treatment in the future. In summary, the automatic method not only reduces the risk of human error during patient setup, improves the efficiency of treatment delivery, but also potentially enhances the patient experiences.

## CONFLICTS OF INTEREST

None of the authors has conflicts of interest or funding to disclose related to the work of this publication.

## AUTHOR CONTRIBUTION STATEMENT

Conceptualization and Methodology: HW, DB, Data Curation: HW, AR, BR, Formal Analysis: HW, TC, Investigation: HW, AR, TC, AM, DB, Supervision: AM, DB, Original draft: HW, All authors have reviewed and edited the manuscript.
